# Prophylactic furosemide to prevent transfusion-associated circulatory overload: a randomized controlled study in rats

**DOI:** 10.1038/s41598-022-16465-z

**Published:** 2022-07-15

**Authors:** Robert B. Klanderman, Joachim J. Bosboom, Denise P. Veelo, Joris J. T. H. Roelofs, Dirk de Korte, Robin van Bruggen, Liffert Vogt, Jaap D. van Buul, Markus W. Hollmann, Margreeth B. Vroom, Nicole P. Juffermans, Bart F. Geerts, Alexander P. J. Vlaar

**Affiliations:** 1 Department of Intensive Care, Amsterdam , UMC-AMC, University of Amsterdam, Amsterdam, The Netherlands; 2 Laboratory of Experimental Intensive Care and Anesthesiology, Amsterdam UMC-AMC, University of Amsterdam, Amsterdam, The Netherlands; 3 Department of Anesthesiology, Amsterdam , UMC-AMC, University of Amsterdam, Amsterdam, The Netherlands; 4 Department of Pathology, Amsterdam UMC-AMC, University of Amsterdam, Amsterdam, The Netherlands; 5 Department of Product and Process Development, Sanquin Research and Landsteiner Laboratory, Amsterdam, The Netherlands; 6 Department of Blood Cell Research, Sanquin Research and Landsteiner Laboratory, Amsterdam, The Netherlands; 7 Department of Nephrology, Amsterdam UMC-AMC, University of Amsterdam, Amsterdam, The Netherlands; 8 Department of Molecular Hematology, Molecular Cell Biology Lab, Sanquin Research and Landsteiner Laboratory, University of Amsterdam, Amsterdam, The Netherlands

**Keywords:** Preclinical research, Respiratory signs and symptoms

## Abstract

Transfusion-associated circulatory overload (TACO) is the leading cause of transfusion related morbidity and mortality. The only treatment is empirical use of furosemide. Our aim was to investigate if furosemide can prevent TACO. A randomized controlled trial was performed using a previously validated two-hit rat model for TACO. Volume incompliance was induced (first hit) in anemic, anesthetized Lewis rats. Rats were randomized to placebo, low-dose (5 mg kg^−1^) or high-dose (15 mg kg^−1^) furosemide-administered prior to transfusion (second-hit) and divided over two doses. Primary outcome was change in left-ventricular end-diastolic pressure (∆LVEDP) pre- compared to post-transfusion. Secondary outcomes included changes in preload, afterload, contractility and systemic vascular resistance, as well as pulmonary outcomes. Furosemide treated animals had a significantly lower ∆LVEDP compared to placebo (p = 0.041), a dose–response effect was observed. ∆LVEDP in placebo was median + 8.7 mmHg (IQR 5.9–11), + 3.9 (2.8–5.6) in the low-dose and 1.9 (− 0.6 to 5.6) in the high-dose group. The effect of furosemide became apparent after 15 min. While urine output was significantly higher in furosemide treated animals (p = 0.03), there were no significant changes in preload, afterload, contractility or systemic vascular resistance. Furosemide rapidly and dose-dependently decreases the rise in hydrostatic pulmonary pressure following transfusion, essential for preventing TACO.

## Introduction

Transfusion-associated circulatory overload (TACO), occurring in up to five percent of transfused patients in the ICU, is the leading cause of major morbidity and mortality following transfusion^1,2^. Furosemide, a loop diuretic, is currently the only treatment available and its use is at best empirical.

TACO is hallmarked by hydrostatic pulmonary edema following transfusion, analogous to acute heart failure (AHF). Considering the similarities, treatment for AHF has been extrapolated to TACO, including diuretics as first-line therapy. However, increasing evidence suggests that TACO has a different etiology compared to conventional volume overload. First, TACO incidence decreased following institution of universal leukoreduction^3^ and up to a third of cases develop fever^4,5^, suggesting an immunological component. Furthermore, the incidence of TACO varies per blood product transfused, suggesting more than pure volume overload^6^. Finally, our previous rat study showed that hydrostatic pulmonary capillary pressure (P_cap_) increased after a red blood cell (RBC) transfusion and not crystalloids^7^. In the ICU, RBC transfusion is associated with an increased P_cap_ and an increased mortality^8^. It is unclear why blood transfusion increases P_cap_, and through what mechanisms cardiac performance and hemodynamics are altered aside from the volume infused.

Prophylactic use of furosemide to prevent TACO warrants further investigation since a lack of evidence precluded the latest meta-analysis to provide any recommendations^9^. The few studies investigating furosemide in the context of transfusion, were performed over 35 years ago. Moreover, whole blood was transfused, and patients included were young with severe anemia due to tropical infections and malnutrition^10,11^. A recent pilot RCT included 80 patients randomized to receive either 20 mg of furosemide or placebo prior to transfusion, leading up to a larger trial^12^. One case of TACO was reported in each arm. There are currently no pre-clinical data on furosemide in the transfusion setting and translational research is essential to guide further clinical studies.

The goal of this study was to assess the effect of furosemide on hydrostatic pulmonary capillary pressure. We hypothesize that furosemide prophylaxis will rapidly decrease P_cap_, and that this effect is dose dependent.

## Results

A total of 18 animals were included in one of three groups (n = 6) randomized to receive low-dose furosemide (5.0 mg kg^−1^), high-dose furosemide (15.0 mg kg^−1^) or placebo. Two rats died prior to randomization: one due to a hemorrhage from the left-internal mammary artery, damaged during thoracotomy and one due to acute cardiac failure following LAD ligation—both were replaced. Additionally, one animal was excluded and replaced post-randomization after autopsy failed to show an MI, thereby lacking a first hit.

### Anemia and volume incompliance

Following isovolemic anemia, hematocrit fell from 42.5 (IQR 39–43) to 31.0 (IQR 29–30.8; p = 2.04 × 10^–4^). Cardiac function decreased following MI: stroke work decreased by − 1462 (IQR − 2590 to 247.7; p = 0.038) and − dP/dt, diastolic relaxation rate, slowed by + 1313 (IQR 271–1827; p = 2.81 × 10^–3^). Ejection fraction was not a reliable variable due to conformational changes of the ventricle following LAD ligation. The MI volume, as surrogate measure of first hit severity, was comparable between groups (Table 1).

**Table 1:** Characteristics at randomization.

Pre-randomization Characteristics	Placebo	Furosemide Low-dose	Furosemide High-dose
Weight (g)	335 (323–343)	354 (347–360)	331 (321–349)
P/F-ratio	398 (388–449)	401 (396–420)	427 (400–489)
LVEDP (mmHg)	12.5 (10.8–13.9)	12.2 (8.8–16.5)	13.4 (12.8–17.0)
Heart rate (min^−1^)	244 (233–249)	252 (241–261)	251 (238–255)
MAP (mmHg)	62 (59–65)	61 (59–63)	63 (61–67)
LVP_max_ (mmHg)	99 (95–110)	101 (99–102)	97 (92–100)
SV (μL)	76 (65–94)	83 (67–95)	69 (61–79)
Ejection fraction (%)	49 (41–67)	59 (58–64)	42 (37–49)
MI size (%)	38.7 (35.7.–41.5)	34.6 (32.0–34.7)	37.6 (34.6–40.3)
RPP (mmHg min^−1^ 10^3^)	25.6 (23.0–26.9)	24.7 (23.8–26.3)	24.4 (23.7–25.7)
Stroke work (mmHg μL 10^3^)	7.1 ± 1.9	7.1 ± 1.5	5.6 ± 1.3
CVP (mmHg)	5.1 (4.0–6.0)	4.24 (4.0–6.0)	6.1 (5.9–6.6)
SVR (dyn s cm^−5^)	251 (201–278)	213 (186–264)	264 (224–335)
Noradrenaline (μg kg^−1^)	0.96 ± 0.16	0.91 ± 0.16	0.79 ± 0.10
Fluid balance (mL)	2.6 ± 0.2	2.9 ± 0.3	2.3 ± 0.2

Data presented as median (IQR) or mean ± SD.

### Effect of furosemide on hydrostatic pulmonary capillary pressure

**Figure 1:**
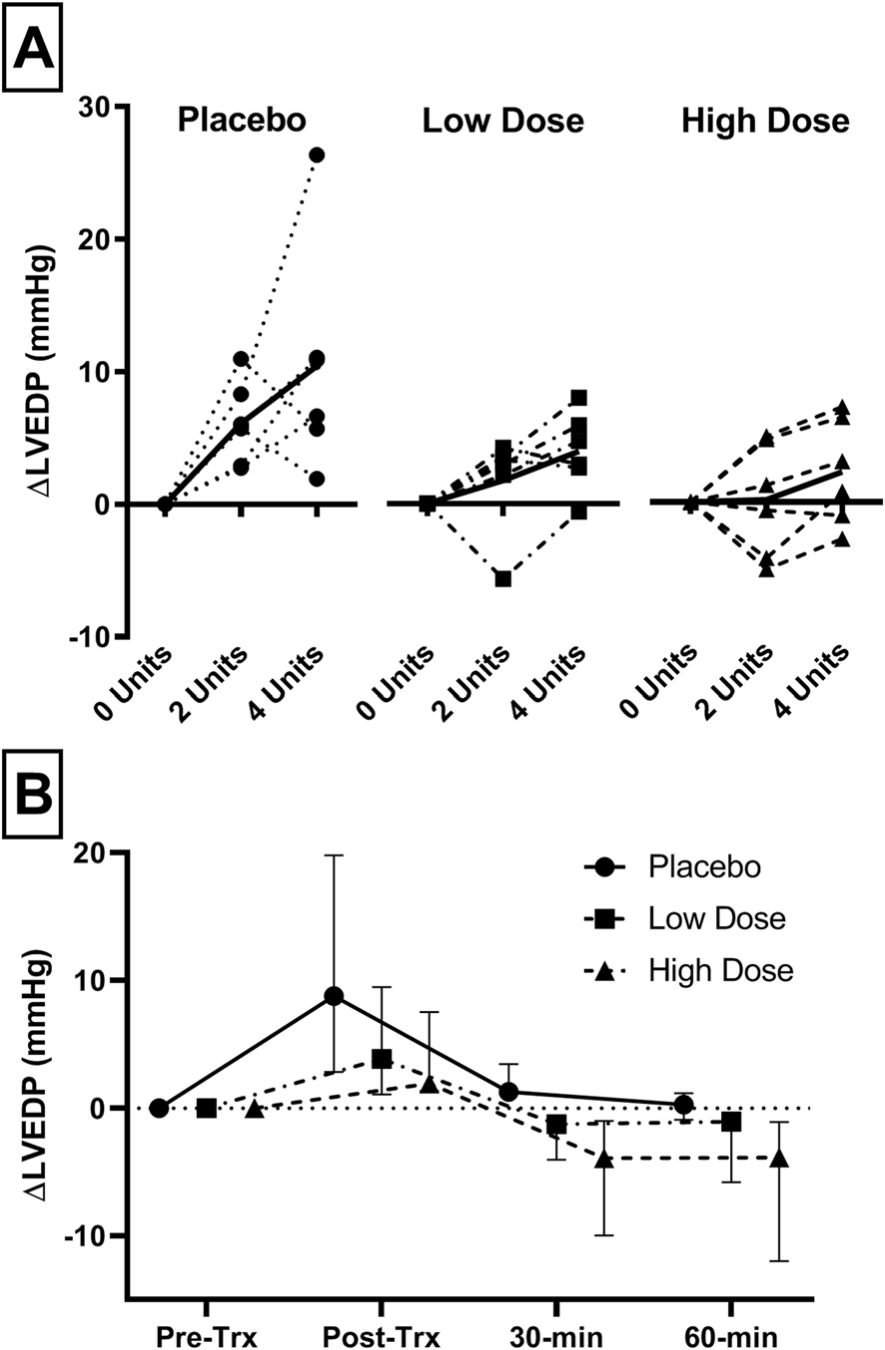
Effect of furosemide on LVEDP. (**A**) The change of LVEDP over the course of the transfusion is given per animal. Animals receiving the placebo group show the greatest increase in ΔLVEDP compared to low and high-dose furosemide. The decrease in LVEDP is dose-dependent and is apparent within 15-min. (**B**) The change of LVEDP over the course of the experiment. Animals receiving placebo have a greater increase in LVEDP post-transfusion and this remains higher through-out the follow-up period. *ΔLVEDP* difference between LVEDP at timepoint compared to baseline, *Trx* transfusion.

Furosemide treatment resulted in a significantly lower ∆LVEDP compared to placebo following transfusion (p = 0.041, Fig. 1). Rats that received furosemide (any dose) had a lower ∆LVEDP after 15 min, following two units of RBCs (p = 0.024), as well as after four units transfused (p = 0.041). Directly following transfusion ΔLVEDP was increased by + 8.7 mmHg (IQR 5.9–11.0) in the placebo group, + 3.9 (IQR 2.8–5.6) in the low-dose group and by + 1.9 (IQR − 0.6 to 5.6) in the high-dose group. At 60 min post-transfusion the furosemide treated group still had a lower LVEDP, though this was not significant (p = 0.103, Fig. 1B).

### Effect of furosemide on preload, afterload, contractility, SVR and electrolytes

**Figure 2:**
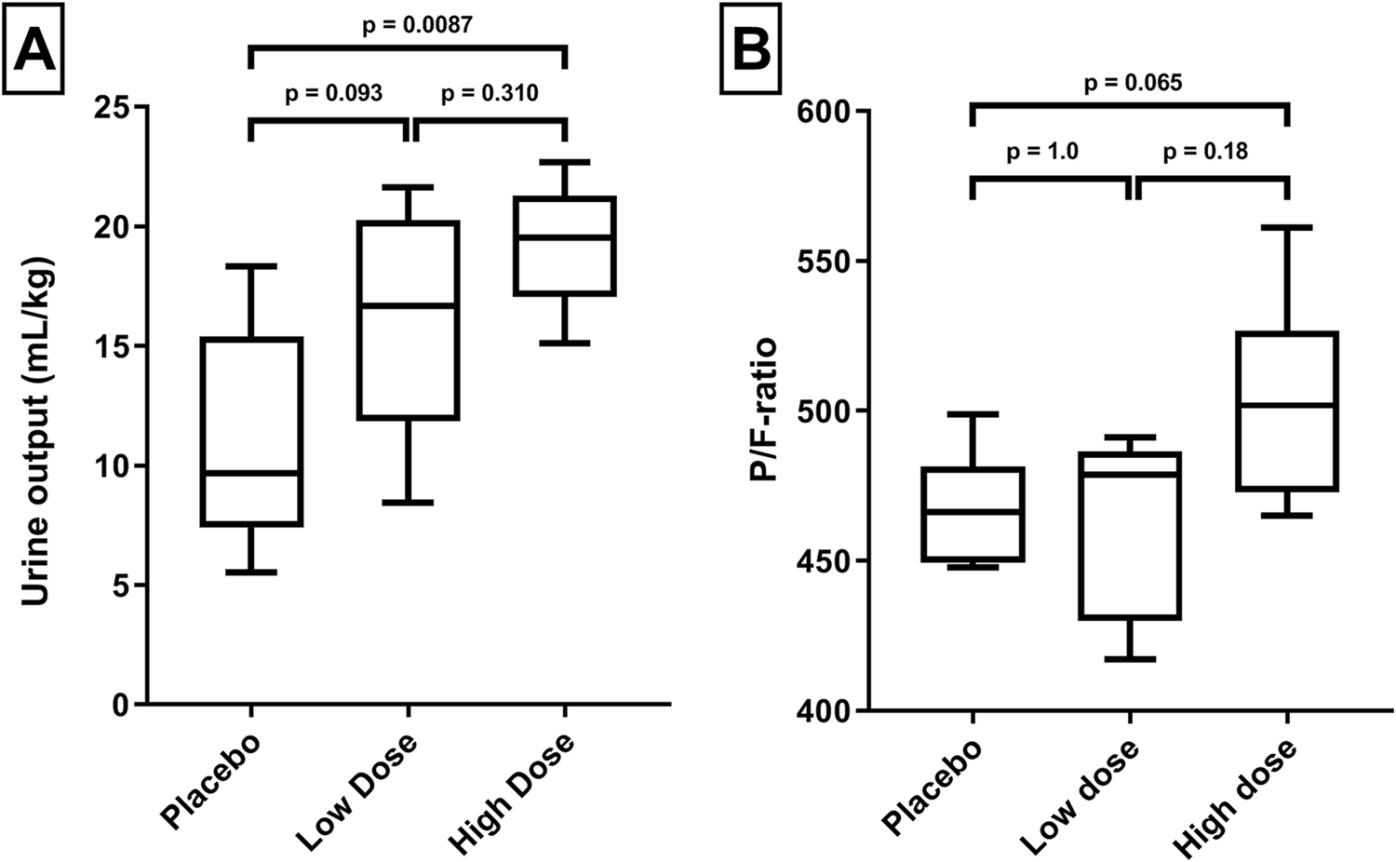
Urine output and P/F-ratios per treatment group. (**A**) There is a dose–effect response to the administration of furosemide and total urine output following randomization. (**B**) P/F-ratio shown between treatment groups.

Preload was assessed by left-ventricular EDV, as well as surrogate markers including urine output, fluid balance, and CVP. Rats receiving furosemide had a significantly increased urine output (mL kg^−1^) compared to placebo (p = 9.70 × 10^–4^), with a dose–response relationship seen (Fig. 2A). Urine output between low-dose and high-dose furosemide was neither significantly different directly post-transfusion (p = 0.94), nor between groups comparing overall urine output (p = 0.59). While the volume of fluid infused was equal between groups (supplementary eTable 1), fluid balance was significantly lower due to a diuresis in rats receiving furosemide + 2.2 mL (IQR 1.2–3.0) versus + 5.2 mL (IQR 3.6–5.7, p = 0.013) in the placebo group. EDV did not differ significantly between placebo, low or high-dose furosemide post-transfusion (Table 2) or after 1-h follow-up (p = 0.372). Further post-hoc analyses of EDV when corrected for HR, LVEDP, SV or CO showed no significant difference. CVP was not different between groups (p = 0.738). There were no significant differences between group sodium and potassium concentrations. Only chloride concentrations were significantly lower in the furosemide treated groups (supplementary eTable 2, p < 0.01).

**Table 2:** Hemodynamic change pre vs. post-transfusion.

Hemodynamic variables:	Placebo	Furosemide low-dose	Furosemide high-dose	p value
∆LVEDP (mmHg)	+ 8.77* (5.94 to 11.02)	+ 3.85 (2.79 to 5.61)	+ 1.93 (− 0.57 to 5.59)	0.103
∆HR (bpm)	+ 5.1 (2.1 to 33.2)	+ 7.9 (4.4 to 27.6)	+ 16.2 (− 5.3 to 27.0)	0.777
**Preload**				
Urine output (mL kg^−1^)	3.01** (0.64 to 4.18)	7.39 (5.18 to 10.26)	8.35 (6.49 to 10.0)	0.013
∆EDV (μL)	− 73.6 (− 95.3 to − 73.6)	− 70.7 (− 80.8 to − 44.5)	− 72.0 (− 87.7 to − 52.7)	0.855
∆CVP (mmHg)	0.32 (− 0.2 to 0.7)	0.2 (− 0.0 to 0.6)	0.0 (− 0.5 to 0.5)	0.738
**Afterload**				
∆MAP (mmHg)	33.1 (23.1 to 42.8)	22.5 (10.9 to 33.0)	33.0 (21.9 to 34.5)	0.372
∆LVP_max_ (mmHg)	22.4 (18.1 to 33.2)	19.1 (15.4 to 27.7)	24.6 (20.9 to 29.1)	0.777
∆BP_Sys_ (mmHg)	22.8 (13.5 to 33.2)	15.4 (12.3 to 27.0)	22.2 (15.2 to 25.6)	0.796
∆BP_Dia_ (mmHg)	40.2 (30.4 to 49.4)	26.8 (15.5 to 36.0)	35.5 (9.8 to 39.7)	0.194
**Contractility**				
∆SV (μL)	− 15.2 (− 36.4 to − 3.6)	− 22.1 (− 29.9 to − 14.3)	− 16.7 (− 29.5 to − 2.43)	0.778
∆RPP (mmHg min^−1^ 10^3^)	7.0 (6.1 to 8.6)	7.5 (5.7 to 8.1)	9.0 (4.7 to 10.0)	0.895
∆Stroke work (mmHg μL)	− 2014 (− 4106 to − 802)	− 2638 (− 3530 to − 1480)	− 1519 (− 3121 to − 500)	0.717
∆dP/dt (mmHg s^−1^)	1966 (451 to 3258)	1614 (1336 to 2427)	2250 (1392 to 3124)	0.864
∆− dP/dt (mmHg s^−1^)	− 2365 (− 3504 to − 435)	− 2309 (− 3930 to − 1803)	− 2574 (− 3741 to − 2143)	0.895
**Vascular resistance**				
**∆**SVR (dyn s cm^−5^)	152.6 (119 to 226)	174.5 (110 to 197)	237.1 (169 to 266)	0.194

Data presented as median (IQR) or mean ± SD. *Significant difference (p < 0.05) placebo vs. furosemide treatment (any dose). **Significant difference (p < 0.01) placebo vs. furosemide treatment (any dose).

Afterload variables included MAP, LVP_max_, systolic and diastolic blood pressure, all of which were not different between groups. SVR also did not differ between groups comparing pre- to post-transfusion and also not after a full hour of follow-up. Contractility reflected by RPP, stroke work, dP/dt and diastolic relaxation (− dP/dt) also did not differ between groups at any time-point.

### Pulmonary outcomes

A higher P/F-ratio was seen in the high-dose group, though this did not reach significance (Fig. 2B). Wet-dry ratio did not differ between furosemide treatment and placebo groups 4.55 (IQR 4.54–4.79) versus 4.53 (IQR 4.42–4.73). Pathology scores were not significantly lower in the furosemide treated groups (p = 0.217).

## Discussion

TACO is associated with severe morbidity and mortality and considering the number of transfusions, prevention of TACO is important. We evaluated prophylactic use of furosemide on P_cap_ in a rat transfusion model. The main findings of this study are (1) furosemide significantly reduces the P_cap_ increase caused by RBC transfusion; (2) there is a dose dependent, early non-diuretic effect of furosemide on LVEDP, apparent within 15 min following administration; (3) left-ventricular preload, afterload, contractility and SVR did not differ between groups.

Most knowledge concerning furosemide is based on AHF research. Since circulatory overload is triggered sooner after transfusion then following conventional fluids^13^, it is unclear whether furosemide would be comparably effective in TACO as in AHF. Furosemide’s actions are divided into an early non-diuretic effect and a natriuretic effect that persists for several hours. The early non-diuretic effect decreases P_cap_ in AHF prior to diuresis, as this effect persists in animal models when the ureters are ligated^14,15^. Supposedly this effect is the result of endothelial prostaglandin synthesis influencing vascular tone^16^, specifically vasodilation of the systemic circulation^17^. Prostaglandin synthesis inhibitors seem to abolish the early non-diuretic effect of furosemide^18,19^. The diuretic effect results from a blocking of the Na^+^-K^+^-2Cl^−^ symporter (NKCC) in the kidney, leading to natriuresis and a decrease in circulating volume. However, NKCC is also located in the lungs on the membrane of alveolar type I and II cells, is involved in alveolar fluid secretion, and is sensitive to loop diuretics^20^. Blocking of the pulmonary NKCC may be an additional target to prevent additional pulmonary edema from forming.

The main finding of this study is that prophylactic use of furosemide significantly reduces the increase of ∆LVEDP following transfusion (Fig. 1). A low hydrostatic pressure by definition cannot result in hydrostatic pulmonary edema and TACO. Already at 15 min there was a dose-dependent LVEDP reduction, while there was no significant difference in urine output post-transfusion between low and high dose furosemide. This underlines that the early non-diuretic effect can blunt an increase in P_cap_ following transfusion, similar to the treatment of AHF^17,21^. This suggests that diuretic prophylaxis directly pre-transfusion may still be effective, while reducing circulating volume through diuresis requires time. Moreover, a lower dose of loop diuretics may already be sufficient in blunting a P_cap_ increase.

In a small survey, clinicians treatment goals in TACO differed from lowering circulating volume or decreasing preload to decreasing afterload^22^. Interestingly, there was no significant effect on preload (including CVP), afterload, contractility or systemic vascular resistance variables between groups. We hypothesize that an increased pulmonary venous capacitance explains why P_cap_ significantly decreases prior to a change in circulating volume. While Dikshit et al.^17^ demonstrated that furosemide increases systemic venous capacitance, our study specifically revealed no lowering of CVP in combination with a decreased EDV. The constant EDV could suggest a guaranteed preload by increased capacitance of the pulmonary capillary bed.

A mechanistic study in isolated dog lungs, showing a rapid increase of pulmonary blood flow following furosemide suggests a decreased pulmonary vascular resistance (PVR) which supports our hypothesis^23^. Unfortunately, PVR is difficult to measure in-vivo in rats, as it requires a pulmonary artery catheter. An increased pulmonary capillary capacitance would increase total pulmonary blood volume (PBV). Seemingly contradicting evidence includes a decrease in PBV shown measured by transpulmonary thermodilution^24^, however this method cannot specifically estimate volumes of different compartments. While our model measured lung wet-weight, this was after exsanguination and can therefore not be used to approximate PBV.

While our study has several strong points, there are a number of limitations. First, the total urine output was not significantly different, while the ΔLVEDP was different between low and high-dose furosemide. In our experiment the diuretic effect might already be near maximal at low-dose, explaining the lack of difference in urine output, while the early non-diuretic effects increase with increasing dose. Furthermore, while investigators were blinded to treatment groups, they could not be blinded for urine output. We minimized bias by not allowing any adjustments to fluid infusion, vasopressors or ventilatory parameters following randomization, which limited the possible follow-up duration of the experiment. Electrolyte fluctuations were not clinically relevant in this animal model however, due to the duration of follow-up might not have become clinically relevant yet. Baseline electrolyte disturbances in critically ill patients can potentially further derail and have deleterious consequences. Finally, the experiment included healthy rats with intact kidney function. Whether the early non-diuretic effect is equally effective in reducing P_cap_ when renal function is impaired, and if dosage changes are required, remains to be determined.

Our results should encourage human trials that assess the preventive effect of furosemide in the context of TACO. So far, prophylactic furosemide is employed in up to 30% of patients transfused^2,25^. If proven beneficial, then more widespread prophylaxis could curb the incidence of TACO. Considering the pharmacodynamics of furosemide, the prophylactic effects likely carry-over in the post-transfusion period. Aside from prevention, its role in the treatment of TACO should be investigated. Additional research is required as to how renal function impacts the effect of furosemide prophylaxis, since renal insufficiency is a major risk factor for TACO and influences furosemide’s effectiveness. Based on this study the low-dose already showed significant reduction in LVEDP, therefore high-dose furosemide might not be required in patients.

In conclusion, our randomized controlled rat study, the prophylactic use of furosemide successfully and rapidly limited the increase in P_cap_ following transfusion. The effect on P_cap_ showed a dose–response relationship to furosemide administration.

## Methods

Animal experiments were approved by the Dutch national committee for animal experiments (Centrale Commissie Dierproeven-*The Hague, The Netherlands*) under license: AVD118002017814. Experiments were separately approved by the center’s animal ethical committee (Dier Ethische Commissie, Academisch Medische Centrum-*Amsterdam, The Netherlands*). Experiments were in accordance with The Guide for the Care and Use of Laboratory Animals^26^. Results are reported according to the ARRIVE 2.0 guidelines.

### Animal protocol

A validated and previously published rat model for TACO was used^7^. Male adult Lewis rats (LEW/SsNHsd, Envigo-*USA*) weighing 300–360 g, were used after a 7-day minimum acclimatization period. General anesthesia was induced by placing animals in a container with 5% isoflurane. After loss of consciousness, a mixture of ketamine (9.0 mg/100 g), dexmedetomidine (12.5 mg/100 g) and atropine (5 mg/100 g) was administered intraperitoneally. A continuous infusion of ketamine (5 mg/100 g h^−1^) was used for maintenance anesthesia over a tail-vein cannula. Rats were mechanically ventilated through a surgically placed tracheal cannula (Babylog 3000, Dräger-*Germany*). Pressure-controlled ventilation was used with tidal volumes of 6 mL kg^−1^ at 60 breaths min^−1^ (guided by PaCO_2_), and 3cmH_2_O positive end-expiratory pressure set. An ultraminiature pressure–volume (PV)-catheter (SPR-838, Millar-*USA*) was retrogradely advanced through the right carotid artery into the left ventricle. The left carotid artery was cannulated to measure arterial blood pressure and for blood sampling with blood gas analyses performed (RapidLab 500, Siemens-*Germany*). A left internal jugular catheter was placed to measure central venous pressure (CVP), administer furosemide or placebo and transfuse RBCs.

Isovolemic anemia was induced through a slow blood draw with 1:1 substitution of colloid (Tetraspan 6%, B. Braun-*Germany*). Substitution was continued until a hematocrit (Htc) of 30% was achieved. Animals were allowed to stabilize for the following 15 min.

A norepinephrine infusion was started targeting a mean arterial pressure (MAP) of 65 mmHg and a left-anterior thoracotomy was performed between the fourth and fifth rib. An acute MI was induced by ligation the left-anterior descending (LAD) coronary artery ± 2 mm below the left-atrium using a 5-0 Prolene suture. The MI was visually confirmed by blanching of the dependent flow territory and ST-elevations on a 3-lead ECG. A 20G chest tube was placed one rib below the incision and the thorax was closed in two layers. Following wound closure, a recruitment maneuver of five breaths at an inspiratory pressure of 25 cmH_2_O was performed and simultaneously the chest tube removed under negative pressure to maximize pulmonary expansion. Rats were allowed to stabilize over 30 min following the MI.

### Randomization, blinding, intervention and transfusion

Randomization was performed after 30 min stabilization using the sealed-envelope method containing the number corresponding to a randomized syringe. Syringes used were identical, prepared under sterile conditions, prefilled prior to the start of the experiments with a similar volume of 600 μL and stored under dark conditions at 20 °C. Placebo syringes contained NaCl 0.9%, high-dose syringes were filled with furosemide 10 mg mL^−1^ (Centrafarm BV*-The Netherlands*) and low-dose syringes were filled with 200 μL of furosemide (10 mg mL^−1^) and 400 μL of NaCl 0.9%. The syringes were randomized using a random number sequence generator (www.random.org/sequences/—accessed March 2018) and stored in ascending numeric sequence guaranteeing a random order.

After randomization, no further adjustments were allowed concerning anesthetic, fluid or vasopressor infusion rates and ventilatory settings to limit confounding of hemodynamic and pulmonary outcomes. Investigators were blinded to the intervention, which was revealed after the inclusion ceased. All rats that completed the protocol were included. Rats that died prior to randomization were replaced. Additional syringes were prepared, and replacement syringes could be requested without loss of blinding.

**Figure 3:**
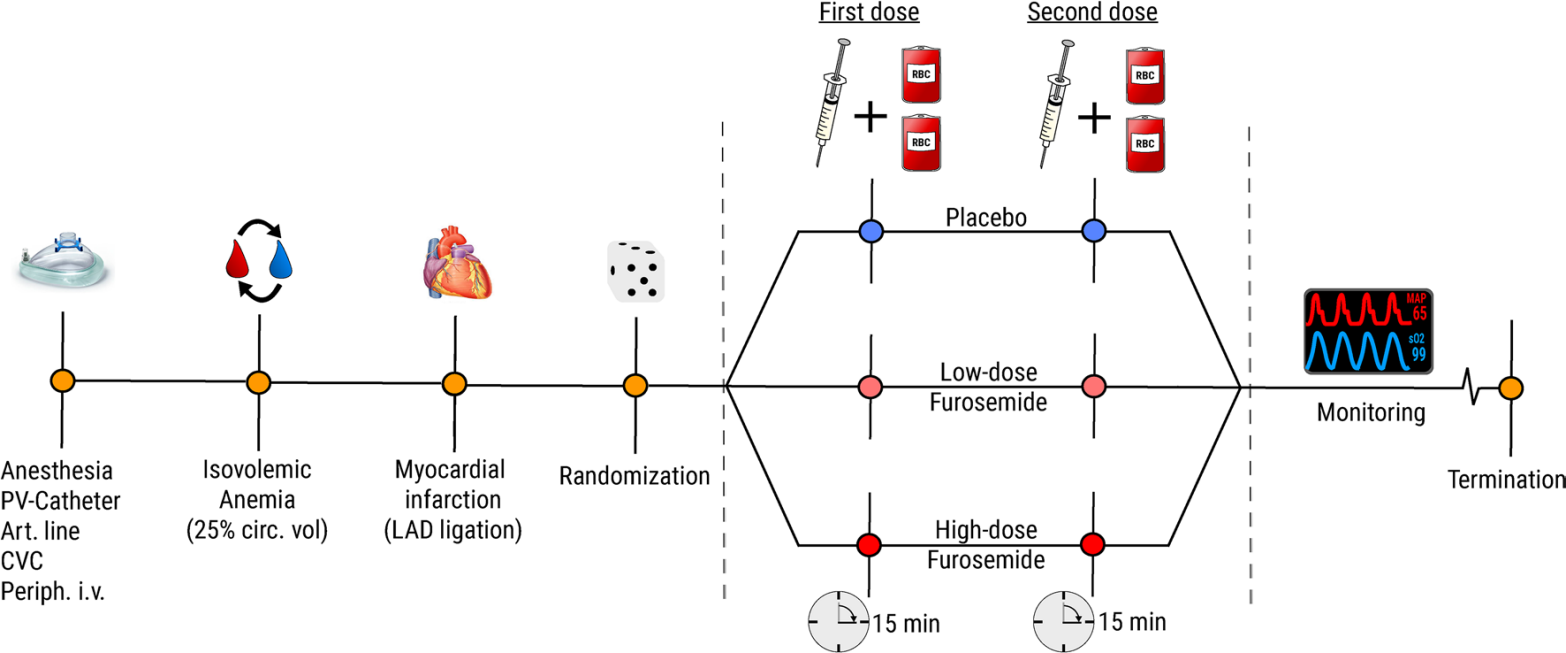
Experimental design. Rats under general anesthesia are made anemic. The first-hit is a myocardial infarction rendering rats volume incompliant. Randomization to placebo, low or high-dose furosemide is performed and prior to transfusion half of the product is administered. Subsequently two RBC units (2.0 mL) are transfused followed by a subsequent dose and transfusion of the remaining two units (2.0 mL). Rats are monitored up to 1 h post-transfusion. *PV-catheter* pressure–volume catheter, *Art.line* arterial line, *CVC* central venous cannula, *Circ.vol* circulating volume, *LAD* left-anterior descending coronary artery.

The furosemide doses were determined based on a previous rat studies, i.e. 5.0 mg kg^−1^ for low-dose and 15.0 mg kg^−1^ for high-dose^27,28^. Dosages correspond respectively to 40 mg and 120 mg (divided over two administrations) in an 80 kg human male. The total dose administered was calculated per animal based on baseline weight (150 μL/100 g). The total dose was spilt into two separate administrations (Fig. 3). At 30-min post-MI the first dose was given intravenously over two minutes, whereafter the transfusion was initiated. Two units were infused over fifteen minutes using a volumetric pump, subsequently hemodynamic measurements were recorded for one minute. The second dose was given over two minutes, and the remaining two RBC units transfused. The volume of one RBC unit was 1.0 mL and approximately 5% of circulating volume, analogous to humans assuming 6 L of blood and ± 300 mL of volume per RBC unit.

Following the transfusion phase, hemodynamic variables were collected for 60 min after which animals were terminated by exsanguination. Longer follow-up was not possible without changing ventilator settings or anesthetic, fluid and/or vasopressor infusion settings which would have induced bias.

### Data and sample collection and processing

Hemodynamic measurements were averaged over one minute and PV-loop measurements were averaged from ten cardiac cycles. Measurements were performed at fixed-time points: at baseline, randomization (pre-transfusion), after the first half of transfusion (2 units of RBCs), immediately post-transfusion and after 60 min follow-up. Total urine output was calculated by weighing spontaneous diuresis, urine massaged from the bladder pre- and post-transfusion as well as to urine aspirated from the bladder during autopsy. Myocardial infarct volume was quantified according to previously published methods to confirm an MI and assess the severity of the first hit between groups^7^.

After termination the lungs were excised. The right upper lobe was examined by an experienced pathologist and rated on a 0 to 3-point scale of increasing pulmonary edema severity. The right lower lobe was weighed (wet-weight) and dried in a stove at 37 °C; dry-weight was determined when the weight no longer decreased after which the wet-dry weight ratio was calculated.

### Preparation of transfusion products

Buffy coat reduced packed RBC units were prepared from whole blood, which was collected from animals outside of the experimental group. Whole blood was collected from healthy adult Lewis rats, anesthetized with isoflurane, through exsanguination via direct cardiac puncture. Whole blood from was collected in a 10:1 ratio with a citrate–phosphate-dextrose solution, pooled from multiple rats and centrifuged at 2000*g* for ten minutes. Under sterile conditions the plasma and buffy coat were removed. The red cell pellet was resuspended in a saline, adenine, glucose and mannitol solution (SAGM), where SAGM was added until a hematocrit of 60% was achieved. RBCs were used within 5 days and all rats in 1 week received a transfusion from this pooled product.

### Outcomes and measurements

The primary outcome was the difference in ∆LVEDP (pre- to post-transfusion) between treatment groups. Secondary outcomes included ∆LVEDP comparing pre-transfusion to 60-min post-transfusion follow-up, urine output during and post-transfusion as well as electrolyte concentrations. Hemodynamic variables included HR, blood pressure, CVP and systemic vascular resistance (SVR). PV-variables included: left ventricular maximum pressure (LVP_max_), end-diastolic volume (EDV), stroke volume (SV), cardiac output (CO), stroke work (calculated as area within the PV-loop) and rate-pressure product (beats/min LVP_max_). Pulmonary outcomes included oxygenation capacity using PaO_2_/FiO_2_-ratio, wet-dry ratio to quantify pulmonary edema as well as a histopathology score.

### Sample size calculation

The sample size was calculated to find a clinically relevant difference in P_cap_ between placebo and high-dose furosemide. A 4.0 mmHg decrease in LVEDP was deemed significant, similar to previous human studies^29^. Based on previously published results the placebo group was estimated to have a mean ∆LVEDP 8.0 ± 1.5 mmHg^7^. In order to detect a clinically relevant effect using a two-tailed alpha of 0.05 and 80% power, a group size of four was required. The group size was increased by 2 rats to correct for possible dropout.

### Statistical analysis

Data were inspected for normality using histograms and was presented as mean ± SD or median (IQR) where appropriate. The Kruskal–Wallis test was used to compare ∆LVEDP between groups. The secondary outcomes including difference between furosemide treated groups versus placebo were compared using a Wilcox-signed rank test. Post-hoc analysis of EDVs were compared using a linear regression with step-wise correcting for the various covariates. Two-way testing was employed and results were considered significant with a p < 0.05, and in case of univariate linear regression with a p < 0.10.

### Ethical approval

Animal experiments were approved by the Dutch national committee for animal experiments (license: AVD118002017814) and the center’s animal ethical committee. Experiments were in accordance with The Guide for the Care and Use of Laboratory Animals^13^, and reported according to the ARRIVE 2.0 guidelines.

## Supplementary Information

Supplementary Information.

## Data Availability

The datasets used and analysed during the current study are available from the corresponding author on reasonable request.

## Abbreviations

AHF: Acute heart failure
BALF: Bronchoalveolar lavage fluid
CO: Cardiac output
CVP: Central venous pressure
dP/dt: Maximum slope of left-ventricular pressure increase during systole
− dP/dt: Maximum slope of left-ventricular pressure decrease during diastole
EDV: (Left-ventricular) end-diastolic volume
HR: Heart rate
LAD: Left anterior descending (coronary artery)
LVEDP: Left-ventricular end-diastolic pressure
LVP_max_: Maximum pressure generated by the left-ventricle
MAP: Mean arterial pressure
MI: Myocardial infarction
PBV: Pulmonary blood volume
PVR: Pulmonary vascular resistance
P/F-ratio: PaO_2_/FiO_2_ ratio
RBC: Red blood cells
P_cap_: Hydrostatic pulmonary capillary pressure
PV-(catheter): Pressure–volume (catheter)
RPP: Rate pressure product (calculated as HR LVP_max_)
Stroke work: Calculated as area with the pressure–volume loop (mmHg μL)
SVR: Systemic vascular resistance (calculated as (MAP-CVP) Cardiac index^−^^1^)
TACO: Transfusion-associated circulatory overload
WD-ratio: Pulmonary wet weight/dry weight ratio
