# Supplementary material for: Prophylactic furosemide to prevent transfusion-associated circulatory overload: a randomized controlled study in rats
Source: Sci Rep. 2022 Jul 15;12:12127. doi: 10.1038/s41598-022-16465-z (PMC9287390; doi:10.1038/s41598-022-16465-z)
Supplement: Supplementary file 1 — Supplementary Information. [file 41598_2022_16465_MOESM1_ESM.docx]

**PROPHYLACTIC FUROSEMIDE TO PREVENT TRANSFUSION-ASSOCIATED CIRCULATORY OVERLOAD – A RANDOMIZED CONTROLLED STUDY IN RATS**

Robert B. Klanderman*^1-3^, Joachim J. Bosboom^3^, Denise P. Veelo^3^, Joris J.T.H. Roelofs^4^, Dirk de Korte^5^, Robin van Bruggen^6^, Liffert Vogt^7^, Jaap D. van Buul^8^, Markus W. Hollmann^2,3^, Margreeth B. Vroom^1^, Nicole P. Juffermans^1,2^, Bart F. Geerts^3^, Alexander P.J. Vlaar^1,2^

^1^ Department of Intensive Care, Amsterdam UMC – AMC, Univ. of Amsterdam – Amsterdam, The Netherlands.

^2^ Laboratory of Experimental Intensive Care and Anesthesiology, Amsterdam UMC – AMC, Univ. of Amsterdam – Amsterdam, The Netherlands.

^3^ Department of Anesthesiology, Amsterdam UMC – AMC, Univ. of Amsterdam – Amsterdam, The Netherlands.

^4^ Department of Pathology, Amsterdam UMC – AMC, Univ. of Amsterdam – Amsterdam, The Netherlands.

^5^ Department of Product and Process Development, Sanquin Research and Landsteiner Laboratory – Amsterdam, The Netherlands.

^6^ Department of Blood Cell Research, Sanquin Research and Landsteiner Laboratory – Amsterdam, The Netherlands.

^7^ Department of Nephrology, Amsterdam UMC – AMC, Univ. of Amsterdam – Amsterdam, The Netherlands.

^8^ Department of Molecular Hematology, Molecular Cell Biology lab, Sanquin Research and Landsteiner Laboratory, University of Amsterdam – Amsterdam, The Netherlands.

**Supplementary eTable 1.** Hemodynamic change pre-transfusion vs. after one-hour follow-up

| **Hemodynamic variables** | **Placebo** | **Furosemide Low-dose** | **Furosemide High-dose** | **p-value** |
| --- | --- | --- | --- | --- |
| ∆LVEDP (mmHg) | +0.27^*^ (-1.18 – 0.90) | -1.05  (-4.76 – -0.70) | -3.87 (-8.10 – -2.77) | 0.181 |
| ∆HR (bpm) | +21.9 (12.4 – 56.7) | +34.3 (15.4 – 44.6) | +38.6 (15.4 – 44.6) | 0.796 |
| **Preload:** |  |  |  |  |
| Urine output (mL/kg) | 9.67^***^ (8.30 – 13.38) | 16.67 (13.35 – 19.58) | 19.51 (17.89 – 20.72) | 0.024 |
| ∆EDV (μL) | -27.7 (-33.1 – 0.87) | 11.0 (-29.9 – 43.5) | -2.2 (-9.5 – 42.4) | 0.372 |
| ∆CVP (mmHg) | -1.05 (-1.3 – -0.17) | -0.89 (-1.5 – -0.05) | -1.09 (-1.4 – -0.9) | 0.805 |
| Fluid balance (mL) | 5.23^*^ (3.55 – 5.75) | 2.40 (0.98 – 3.89) | 2.18 (1.46 – 2.84) | 0.047 |
| **Afterload:** |  |  |  |  |
| ∆MAP (mmHg) | 18.6 (12.6 – 31.3) | 17.4 (11.1 – 26.6) | 12.2 (2.3 – 16.0) | 0.240 |
| ∆LVP_max_ (mmHg) | 20.9 (12.7 – 21.2) | 19.0 (11.3 – 24.7) | 13.9 (2.6 – 15.7) | 0.372 |
| ∆BP_Sys_ (mmHg) | 14.8 (-1.2 – 31.0) | 12.6 (10.4 – 14.5) | 0.7 (-0.6 – 12.5) | 0.172 |
| ∆BP_Dia_ (mmHg) | 15.5 (11.5 – 36.6) | 18.9 (12.0 – 30.6) | 14.6 (6.1 – 24.3) | 0.688 |
| **Contractility:** |  |  |  |  |
| ∆SV (μL) | -42.1 (-42.6 – -11.0) | -30.7 (-39.4 – 13.8) | -29.1 (-36.5 – -21.5) | 0.587 |
| ∆RPP (mmHg/min•10^3^) | 8.0 (5.6 – 11.0) | 7.7 (7.1 – 9.6) | 7.7 (1.9 – 12.0) | 0.927 |
| ∆Stroke work (mmHg•μL) | -877 (-1547 – -54.1) | -1801 (-2619 – -409) | -1353 (-1626 – -127) | 0.548 |
| ∆dP/dt (mmHg•s^-1^) | 2005 (174 – 3076) | 1590 (729 – 2119) | 1641 (1044 – 2143) | 0.854 |
| ∆-dP/dt (mmHg•s^-1^) | -2562 (-3052 – -1558) | -1764 (-2183 – -1352) | -2141 (-2452 – -1827) | 0.493 |
| **Vascular resistance** |  |  |  |  |
| **∆**SVR (dyn•s/cm^5^) | 251 (158 – 306) | 164 (111 – 358) | 191 (158 – 240) | 0.864 |

Data presented as median (IQR) or mean +/- SD. ^*^Significant difference (p < 0.05) placebo vs. furosemide treatment (any dose). ** Significant difference (p < 0.01) placebo vs. furosemide treatment (any dose). *** Significant difference (p < 0.001) placebo vs. furosemide treatment (any dose).

**Supplementary eTable 2.** Furosemide associated electrolyte changes

| **Sodium (mEq/L):** | **Placebo** | **Furosemide Low-dose** | **Furosemide High-dose** | **p-value** |
| --- | --- | --- | --- | --- |
| Pre-randomization | 134 (133 – 135) | 135 (132 – 135) | 134 (133 – 134) | 0.928 |
| Post-transfusion | 141 (140 – 143) | 142 (141 – 142) | 142 (141 – 142) | 0.851 |
| Termination | 143 (142 – 144) | 143 (142 – 143) | 141 (140 – 143) | 0.318 |
| **Potassium (mEq/L):** |  |  |  |  |
| Pre-randomization | 4.9 (4.9 – 5.2) | 5.5 (5.0 – 5.5) | 5.0 (4.9 – 5.4) | 0.559 |
| Post-transfusion | 4.2 (4.1 – 4.3) | 4.4 (4.1 – 4.4) | 4.0 (3.9 – 4.1) | 0.088 |
| Termination | 4.6 (4.3 – 4.8) | 4.5 (4.4 – 4.7) | 4.2 (4.2 – 4.2) | 0.098 |
| **Chloride (mEq/L):** |  |  |  |  |
| Pre-randomization | 105 (103 – 107) | 104 (104 – 104) | 105 (103 – 105) | 0.985 |
| Post-transfusion | 111 (110 – 111) | 110 (107 – 110) | 108 (108 – 109) | 0.336 |
| Termination | 115 (113 – 116) | 110 (109 – 111) | 111 (109 – 113) | 0.022 |

Data is presented as median (IQR). ^*^Significant difference (p < 0.01) placebo vs. furosemide treatment (any dose).
